# Laser-Aided Directed Energy Deposition of Steel Powder over Flat Surfaces and Edges

**DOI:** 10.3390/ma11030435

**Published:** 2018-03-16

**Authors:** Fabrizia Caiazzo, Vittorio Alfieri

**Affiliations:** Department of Industrial Engineering, University of Salerno, 84084 Fisciano, Italy; valfieri@unisa.it

**Keywords:** Directed Energy Deposition, steel powder, Additive Manufacturing, laser material processing

## Abstract

In the framework of Additive Manufacturing of metals, Directed Energy Deposition of steel powder over flat surfaces and edges has been investigated in this paper. The aims are the repair and overhaul of actual, worn-out, high price sensitive metal components. A full-factorial experimental plan has been arranged, the results have been discussed in terms of geometry, microhardness and thermal affection as functions of the main governing parameters, laser power, scanning speed and mass flow rate; dilution and catching efficiency have been evaluated as well to compare quality and effectiveness of the process under conditions of both flat and edge depositions. Convincing results are presented to give grounds for shifting the process to actual applications: namely, no cracks or pores have been found in random cross-sections of the samples in the processing window. Interestingly an effect of the scanning conditions has been proven on the resulting hardness in the fusion zone; therefore, the mechanical characteristics are expected to depend on the processing parameters.

## 1. Introduction

To prevent replacement of high-price sensitive metal products such as turbine blades, rotor shafts, tools and moulds, Additive Manufacturing is growing [1,2]. The driving idea is to provide additional metal over worn-out surfaces to restore nominal dimensions, thus preventing part disposal.

In this framework, a number of laser-based methods have been employed in demanding environments such as aerospace, defense, petrolchemical and nuclear industries [3,4]. In Directed Energy Deposition (DED), a laser beam is used as a heat source to scan the surface: a melting pool over an existing substrate is created and metal impinging of the pool is fed concurrently (i.e., in single stage processing) [5]. A deposited metal trace results, with metallurgical bonding to the substrate thanks to fusion and diffusion. Resistance metallic coatings can be laid [6]; functionally graded material parts can be manufactured by means of a dual powder feeding system [7]; and the fabrication of a 3D part is even possible by means of layer-by-layer deposition of consecutive, overlapping traces [8]. Reduction of waste is benefited in comparison with subtractive technologies [9]. Different names have been given to the same technology, depending on the Authors or the company [10].

In general, a number of advantages are reported in DED with respect to conventional welding technologies for repairing, such as tungsten inert gas or gas metal arc [5,11]. As focused heat is provided, thermal affection and residual stresses are reduced, and precision and production flexibility are enhanced [3]. New opportunities are hence expected for parts of complex geometry and difficult-to-cut materials [12].

At present, both powder and wire are being considered as feedstock. It is worth noting that metallic wires are considered to be easier to stock and to produce in comparison with powder [13]; on the other hand, process stability, proper surface quality, bonding strength and soundness are reported to be challenging [14]. Since powder feeding has been proven to be flexible in materials [15], as well as robust and effective, several studies in the literature have discussed the outcome as a function of the processing parameters [16,17].

Regarding steel deposition in the form of powder, the influence of the particle size distribution on the resulting relative density has been investigated [18]. Interestingly, since the cost of metal powder is among the main barriers in DED, the feasibility of a raw feedstock consisting of coarse and irregular steel swarf or chips resulting from machining has been proposed [19]. 

Clad thickness, depth of penetration and extent of the heat-affected zone (HAZ) are governed by the main parameters such as the laser power, the scanning speed and the specific energy of the beam [5,6]. In addition, depending on the carbon content and irrespective of the feedstock form [20], martensitic transformation occurs in the base metal [21], i.e., the microstructure morphology in terms of grain size and the mechanical properties in turn [22] have been found to definitely depend on solidification velocity and the temperature gradient as a consequence of the processing parameters [23], mainly the scanning speed [10]. The latter has been reported to significantly influence dilution [3], i.e., the percentage index of the affection of the substrate with respect to the reported metal [11]. Minor porosity and micro-cracking have been observed [6] due to residual thermal stresses, although these are not deemed to result in rejection of parts during quality checks when referring to usual international or customer standards for quality in laser welding [24], since no specific regulations are available at present for DED [25].

It is worth noting that, as for any emerging technology in the industry [26,27], flexible thermal modeling is crucial to prevent the usual trial-and-error approach. Indeed, much effort has been made in the literature to model the process of DED in terms of resulting geometry and induced thermal cycles [28].

Numerous studies have been conducted over flat surfaces. These results are suitable for actual applications in repair process chains, where damaged areas or cracks are preliminarily removed by milling, then reconditioned with new material deposition [29]. Nevertheless, processing of sharp edges is required to be investigated as well, since this processing configuration is expected to be useful in many applications, e.g., for the purpose of overhauling cores and mould sections for investment casting. As a consequence, deposition over both flat surfaces and corners has been considered in this paper.

## 2. Materials and Methods 

A base metal corresponding to standard UNS S43100 chromium-nickel stainless steel in terms of nominal chemical composition (Table 1) has been considered. Pre-alloyed, spherical shaped, gas-atomized virgin commercial powder of the same composition has been used; the particle size ranges from 20 µm to 60 µm. Since a steady feeding rate must be provided consistently [11], the powder has been preliminary dried, in furnace, 180 °C, 2 h, to flow properly via an antistatic conveyor to the working area. 

**Figure 1:**
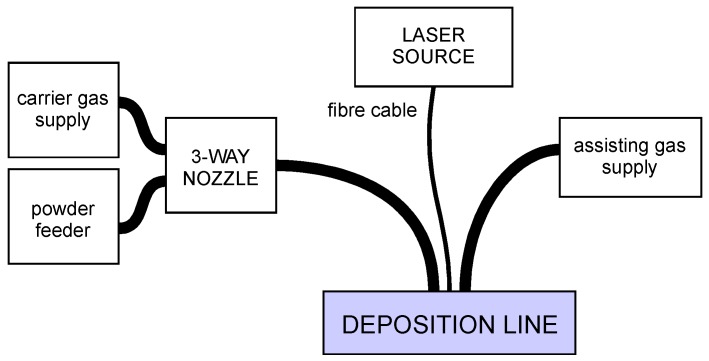
Main components in the deposition line.

**Figure 2:**
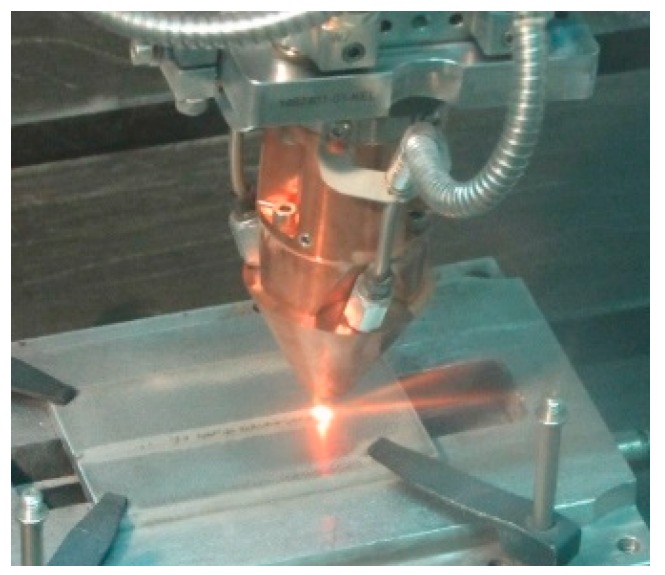
Laser head performing DED over a flat surface.

To perform DED, a deposition line (Figure 1) is required, and a number of base components must be arranged [25], irrespective of the form of the feedstock supply. For the purpose of this work, a fibre-delivered Yb:YAG disc laser source, operating in continuous wave emission (Table 2), has been considered. The movement of the laser head has been accomplished by a 6-axis industrial robot with a dedicated controller, and an in-built 3-way feeding nozzle has been moved with the laser head (Figure 2); the base metal has been provided by a powder feeder with oscillating conveyor.

Argon has been supplied: it has been injected with each stream of powder as a carrier gas at a flow rate of 3 L/min; it has been blown coaxially to the laser beam at a flow rate of 10 L/min as a shielding gas over the melting pool. A tilting angle of 4° has been set for the laser head, in agreement with common practice to process high-reflective metals [30] to prevent back-reflections from entering the optics train. A given positive defocusing providing a processing laser beam diameter of 3 mm on the substrate has been set, thus benefiting from reduced irradiance and increased catchment of powder in the melting pool with respect to a focused condition.

**Figure 3:**
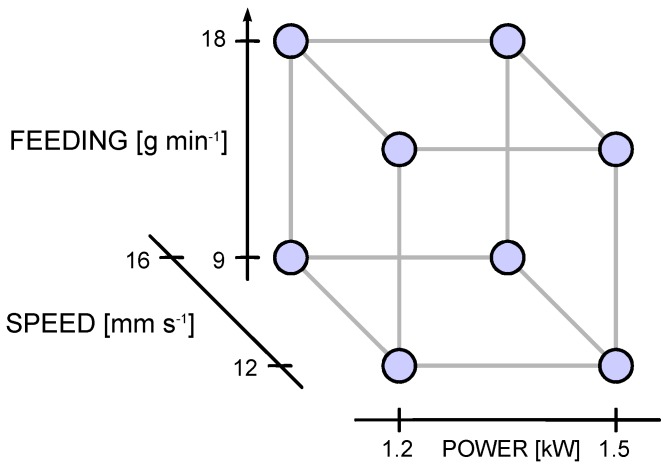
Testing scheme, full-factorial, 3-factor, 2-level plan.

The main processing factors of the experimental plan have been selected based on the literature and past experience: namely, laser power *P*, scanning speed *s* and powder feeding rate *m*′ have been considered. Two levels for each factor have been set upon preliminary trials and adjusted to result in valuable outcomes, preventing detachment, balling, lack of clad or excessive dilution. A full-factorial, 3-factor, 2-level experimental plan has been arranged (Figure 3); eight deposition conditions have resulted (Table 3) and have been performed over both flat surfaces and edges. A total deposition length of 100 mm has been set. A 0.3 mm beveled edge has been produced by machining prior to deposition to resemble an actual condition of wear. Three runs have been planned for each processing condition in random order. The geometry has been evaluated upon cross-cutting and mechanical preparation; three samples from each trace have been considered, the results have been averaged to assess the statistical significance. Polishing to mirror finish and chemical etching with a solution consisting of 5% nitric acid and 95% ethanol at room temperature [31] have eventually been performed.

**Figure 4:**
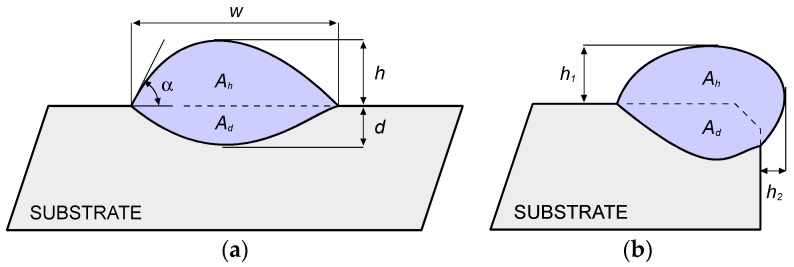
Scheme of the cross-section resulting from deposition (**a**) over flat surface and (**b**) edge.

In agreement with common practice in DED, a number of geometrical responses in the cross-sections have been measured using conventional optical microscopy. Namely, in case of deposition over flat surfaces (Figure 4a): width *w*, height *h*, depth *d*, shape angle α at both sides of the trace, areal extent *A_h_* and *A_d_* of the deposition above and below the reference plane, respectively. In case of deposition over edges (Figure 4b): height *h*_1_ and *h*_2_ with respect to the reference planes, areal extent *A_h_* and *A_d_* of the deposition outside and inside the theoretical edge, respectively.

Dilution has been considered as well: Although a chemical definition is usually given involving the weight percentage composition of the main alloying elements in the substrate with respect to the reported metal [11], it has been shown that an alternative geometric definition can be given [32]; namely, the ratio of the fused (i.e., mixing) area of the substrate to the total area in the transverse cross-section can be measured. Therefore, dilution in this paper has been evaluated as:

(1) $$dilution=\frac{A_{d}}{A_{h}+A_{d}}\left[\%\right]$$

Moreover, the catching efficiency η has been rated as a measure of the process effectiveness [11]: the ratio of the deposited metal *m_d_* to the total amount of delivered powder *m_t_* in the processing time has been evaluated. The higher this ratio, the better the efficiency, since a larger amount of powder is caught by the fused pool. 

In practice, the mass of deposited metal has been found by mere weighing of the samples, whereas the total mass of delivered powder has been found based on the flow rate *m*′ and the deposition time *t_d_* to provide the trace length *L*:

(2) $$\eta =\frac{m_{d}}{m_{t}}=\frac{m_{d}}{m^{\prime }\times t_{d}}=\frac{m_{d}}{m^{\prime }\times \frac{L}{s}}\left[\%\right]$$

Eventually, Vickers microhardness testing has been performed to assess the extent of the HAZ between the fusion zone and base material, as well as to discuss the microstructural changes upon deposition; an indenting load of 0.2 kg has been used for a dwell period of 10 s, a step of 200 μm has been allowed between consecutive indentations, in compliance with the usual ISO standard [33] for hardness testing of metallic materials.

## 3. Results and Discussion

### 3.1. Deposition over Flat Surfaces

#### 3.1.1. Geometry

Since all of the processing conditions of the experimental plan resulted in a valuable outcome (Table 4), all of them can be effectively considered and discussed (Table 5); the catching efficiency has been rated as well.

**Figure 5:**
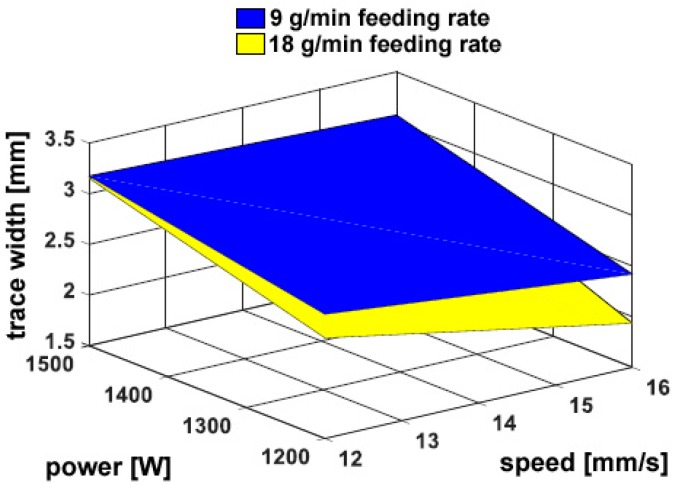
Trace width *w* as a function of the governing variables; flat deposition.

No cracks or pores have been found. Interesting findings can be drawn based on multifactor trends of the geometrical responses. At first, width *w* increases for increasing power and decreasing speed (Figure 5); therefore, any increase in thermal input (i.e., the power to speed ratio) yields a corresponding increase in trace width, in agreement with past research on DED of metals [25]; the shape angle is affected, accordingly. The effect of the feeding rate is not significant with respect to trace width; hence, one may infer the attenuation of the laser beam due to powder blowing [11] is negligible in the investigating domain. 

**Figure 6:**
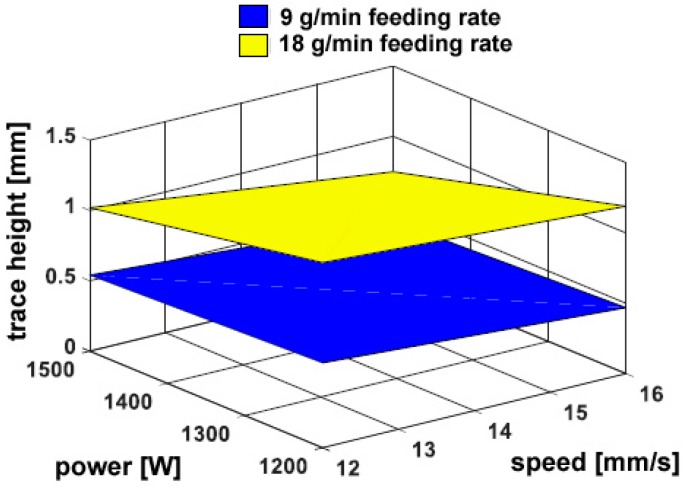
Trace height *h* as a function of the governing variables; flat deposition.

**Figure 7:**
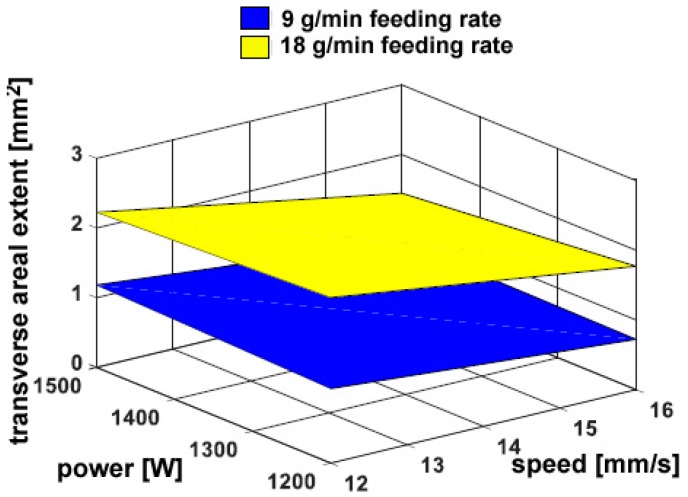
Transverse areal extent of reported metal *A_h_* as a function of the governing variables; flat deposition.

**Figure 8:**
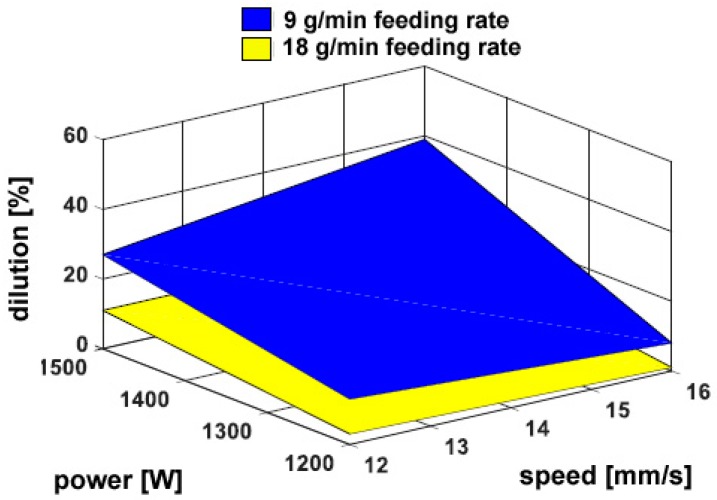
Dilution as a function of the governing variables; flat deposition.

On the other hand, a considerable dependence on the powder feeding rate has been found for both the trace height *h* (Figure 6) and the transverse areal extent of reported metal *A_h_* (Figure 7), as expected: namely, a 50% increase in delivered powder results in the same increase in transverse size of the reported metal; the catching efficiency is not affected. Since the fusion zone below the reference plane is negligible with respect to the fusion zone above, the same trends have been found when considering the total size of fusion area. As an additional consequence, dilution is mainly governed by *A_h_* and rather approaches its inverted trend (Figure 8). It is worth noting that although limited dilution below 10% results in some of the processing conditions, the process is expected to be successful thanks to diffusion bonding [25].

The approximation of certain transverse profiles for the fused area *A_h_* above the reference plane has been addressed in the literature [8,34] to provide assistance to modelling. Namely, an approximation for *A_h_* can be given by a parabolic or circular segment. The effectiveness of these has been further investigated here, based on the real measurements in the cross-section (Table 5). A comparison has been made (Table 6): interestingly, the parabolic approximation is effective in a measure of 5.6% on average, although the circular segment is affected by a 6.4% average absolute mismatch. The result is relevant to real applications: a geometric model, as simple as a parabolic profile, is useful when a processing strategy for consecutive, and overlapping traces must be set to properly scan wider surfaces or manufacture a real part [8].

#### 3.1.2. Micro-Hardness

**Figure 9:**
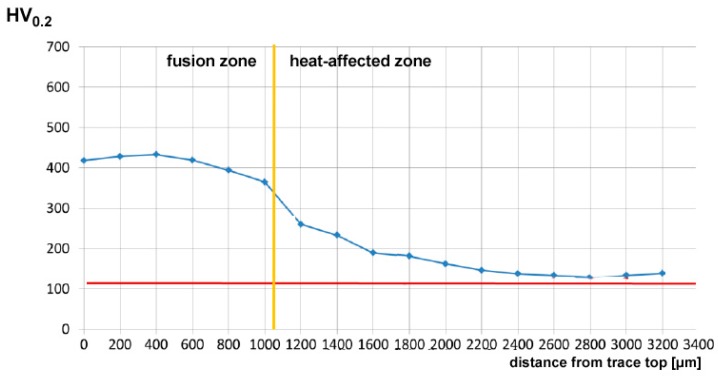
Example of the trend of Vickers micro-hardness under condition F; flat deposition.

In order to give a full description of the outcome upon DED, the mechanical properties have been investigated by means of Vickers micro-hardness testing. All of the processing conditions have been addressed, and random cross-sections have been indented; hence, the trend of micro-hardness has been evaluated as a function of the distance from the top surface of the reported metal (Figure 9). Based on this, the average hardness in two sites of interest (i.e., the reported metal and the HAZ) can be discussed as a function of the processing parameters (Table 7); it is worth noting that larger standard deviations result in the HAZ with respect to the reported metal as a consequence of ranging in a wider window.

**Figure 10:**
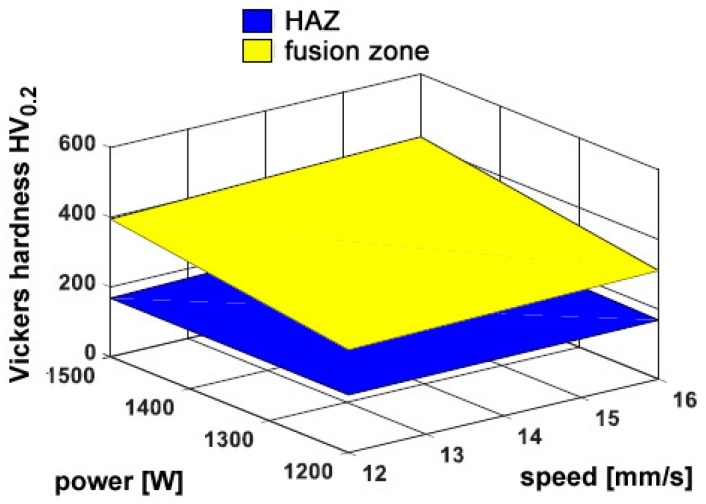
Example of the trend of Vickers micro-hardness in condition F; flat deposition.

Interestingly, hardness in the fusion zone is clearly governed by the processing conditions because the cooling rate is affected, and the resulting microstructure and grain size in turn, in agreement with similar findings in the referred literature [10,22,23]. Indeed, an increase of hardness has been found ranging from 270 HV_0.2_ to 450 HV_0.2_ in the fusion zone, depending on the processing conditions, with respect to a reference 115 HV_0.2_ in the parent metal. Power is deemed to exert the main effect, whereas any dependence on the feeding rate is not significant; as a consequence, the response surfaces for hardness have been averaged for each given condition of power and speed (Figure 10).

**Figure 11:**
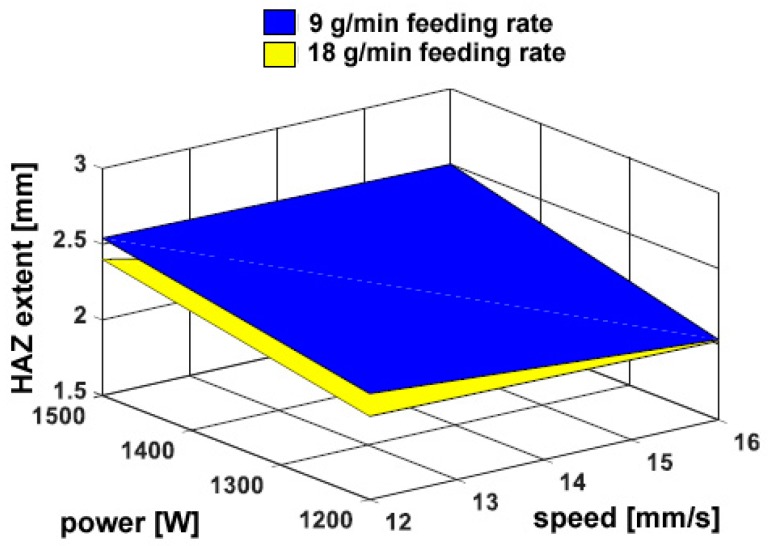
Extent of the HAZ as a function of the processing conditions; flat deposition.

When this approach is taken to draw the response surface, the resulting hardness in the HAZ appears to be unaffected by the processing conditions. Nevertheless, the extent of the HAZ can be inferred (Table 8) based on the distance to recover the reference hardness in the parent metal. As expected, larger HAZs have been found when a higher thermal input is provided (Figure 11), i.e., when a lower processing speed is set. Again, any dependence on the flow rate is weak.

### 3.2. Deposition over Edges

#### 3.2.1. Geometry

All of the processing conditions of the experimental plan resulted in a valuable outcome over edges as well (Table 9). It is worth noting that a proper metal allowance must be provided in order to result in effective addition of metal upon eventual post processing machining when a sharp edge is recovered. The shape in the cross-section and the corresponding dilution and catching efficiency have been considered (Table 10). Due to the peculiar shape of the reported metal in the cross-section, approximations via a parabolic or circular segment profile are prevented. Further studies are planned to find a proper geometric approximation to set the processing path.

**Figure 12:**
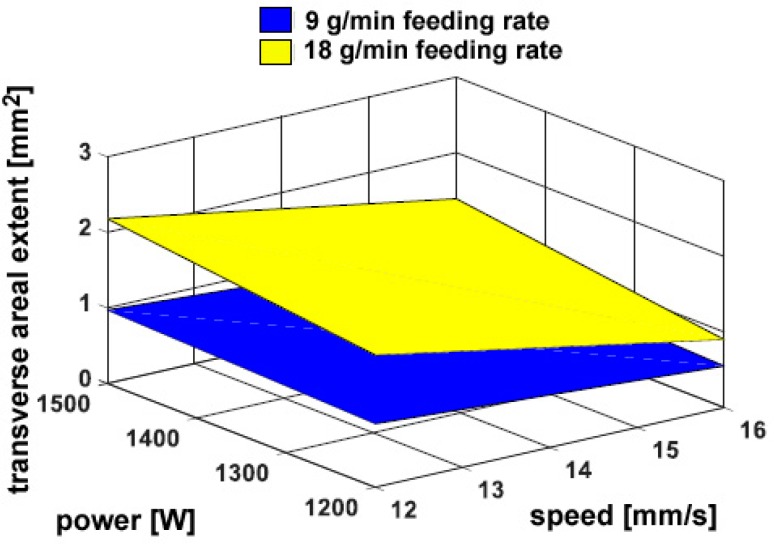
Transverse extent of reported metal *A_h_* as a function of the governing variables; edge deposition.

**Figure 13:**
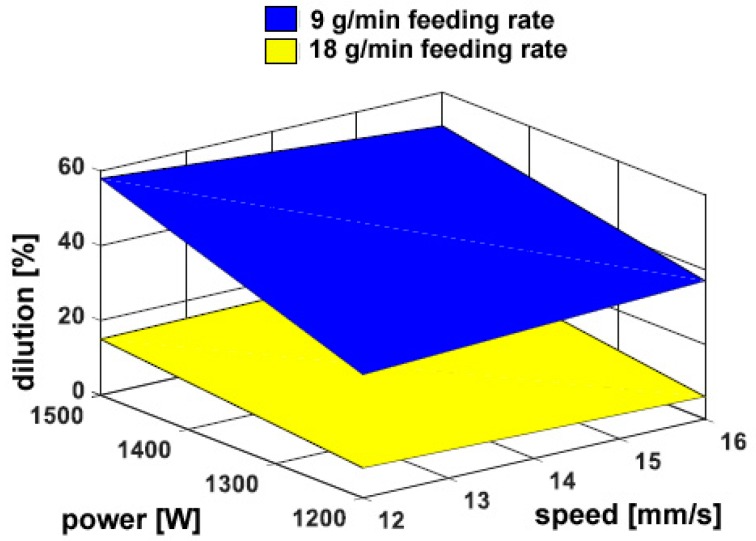
Dilution as a function of the governing variables; edge deposition.

As for deposition over flat surfaces, no cracks or pores have been found. Again, a 50% increase in delivered powder results in a comparable increase in the transverse size of reported metal (Figure 12). The overall geometry depends on the thermal input. Nevertheless, as a consequence of a different shape of the substrate, the catchment is affected: in general, a reduction of 20% on average results in the catching efficiency, as a consequence of the powder flow impinging an edge instead of a flat surface. Conversely, dilution is increased up to 58%, although the same trend is observed (Figure 13), approximately, with respect to deposition over flat surfaces, since the amount of reported metal is reduced due to the edge.

#### 3.2.2. Micro-Hardness

**Figure 14:**
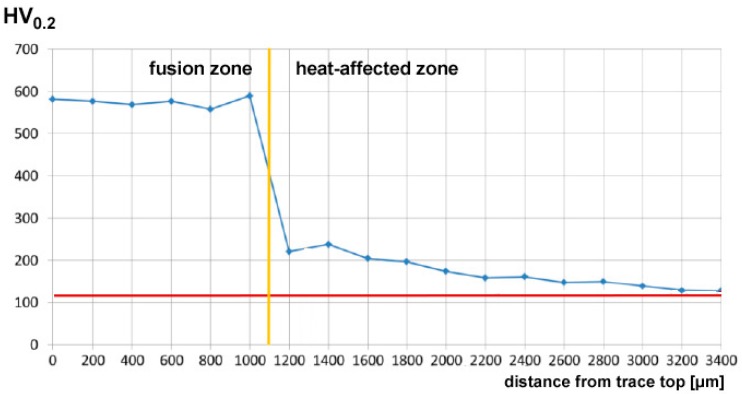
Example of the trend of Vickers microhardness under condition F; edge deposition.

**Figure 15:**
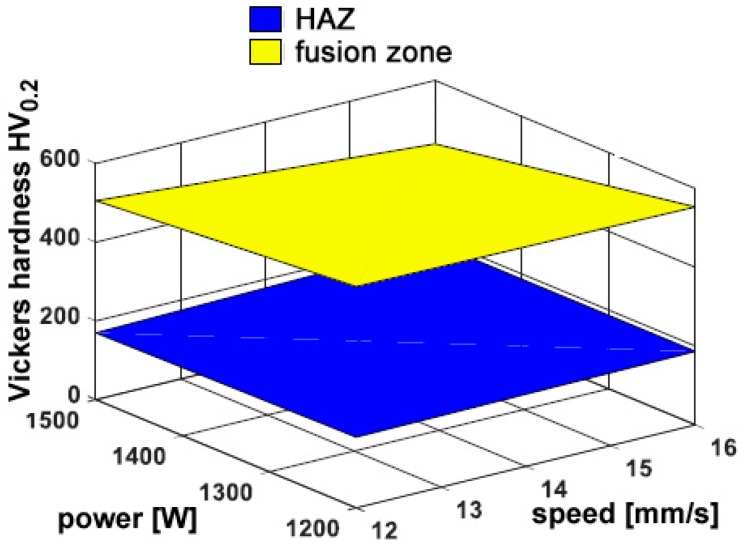
Average Vickers micro-hardness in the deposited metal; edge deposition.

Regarding the mechanical properties of the reported metal, the trend of Vickers micro-hardness has been evaluated along a 45°-tilted path with respect to the theoretical edge, starting from the trace top (Figure 14). Dependence on the processing conditions is considered (Table 11, Figure 15), although higher hardness has been measured for each given processing condition with respect to the same condition of deposition over flat surfaces. Again, one may infer the cooling rate in case of deposition over edges is affected, thus resulting in different microstructure and grain size in the fusion zone. Aimed at to a real application, the proper condition of processing must be selected based on the damage to be addressed; an approach of optimization to take account of a number of requirements, both geometrical and mechanical with specific weights depending on the application, is suggested in the literature [25].

## 4. Conclusions

The effectiveness of the process of Directed Energy Deposition has been addressed over both flat surfaces and edges, aimed at real applications where coating or overhauling for the purpose of restoring the nominal dimensions is required. Convincing findings have been achieved. 

Namely, a proper processing window has been found to perform the process over UNS S43100 chromium-nickel stainless steel. Based on the application, the proper condition must be selected in a multi-constraint optimization approach. Nevertheless, a number of main findings can be given here:

- the approximation of a parabolic profile in the cross-section is suitable in case of flat deposition; hence, a simple description is benefited when setting a proper deposition strategy for overlapping, multi-layer traces at the pre-processing stage;
- no cracks or pores are produced in random cross-sections of the samples in the suggested processing window;
- the catching efficiency depends on the processing parameters; half the amount of the total delivered powder is effectively deposited on average for both flat and edge depositions;
- dilution, hence the affection of the parent metal, is mainly governed by the thermal input and is increased when moving from flat to edge deposition as a consequence of modified thermal exchange.

Tensile and fatigue strengths are expected to depend on the processing condition of deposition. Dependence of the Vickers microhardness on the thermal input has been found.

## Figures and Tables

**Table 1:** Nominal chemical (wt.%) composition of base and fed metals.

Cr	Ni	Mn	Si	C	P	S	Fe
15.0 ÷ 17.0	1.25 ÷ 2.50	1.0	1.0	0.20	0.04	0.03	Balanced

**Table 2:** Main technical features of the laser source.

Parameter	Value
Maximum output power (kW)	4.0
Operating nominal wavelength (nm)	1030
Beam Parameter Product (mm × mrad)	8.0
Core diameter of the delivering fibre (µm)	300
Spot size of the laser beam on the surface (mm)	3.0

**Table 3:** Processing condition of the experimental plan for deposition over flat surface and edges.

Condition	*P* (kW)	*s* (mm∙s^−1^)	*m*′ (g∙min^−1^)
**A**	1200	12	9
**B**	1200	12	18
**C**	1200	16	9
**D**	1200	16	18
**E**	1500	12	9
**F**	1500	12	18
**G**	1500	16	9
**H**	1500	16	18

**Table 4:** Trace surface aspect and corresponding cross-section for deposition over a flat surface.

Condition	Trace Aspect	Cross-Section
**A**	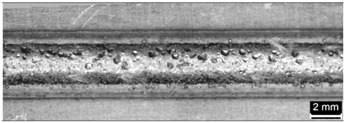	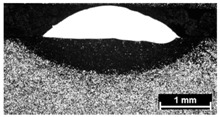
**B**	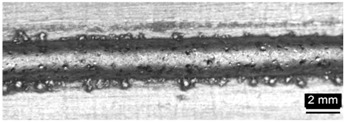	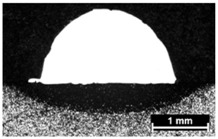
**C**	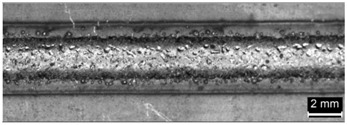	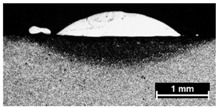
**D**	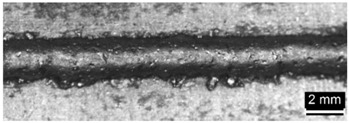	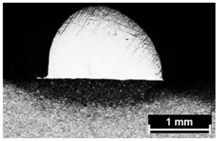
**E**	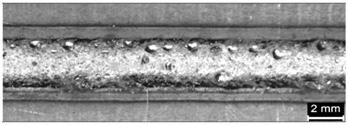	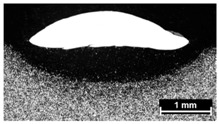
**F**	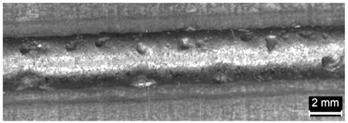	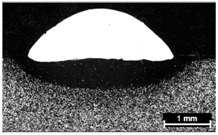
**G**	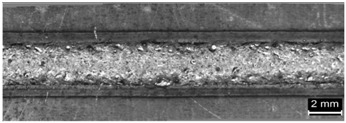	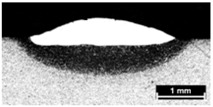
**H**	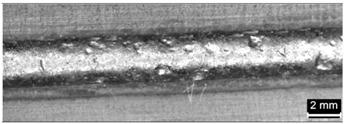	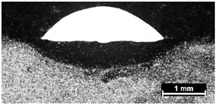

**Table 5:** Average geometrical responses in the cross section for deposition over a flat surface.

Condition	*w* (mm)	*h* (mm)	*d* (mm)	*A_h_* (mm^2^)	*A_d_* (mm^2^)	*α* (°)	Dilution (%)	*η* (%)
**A**	2.73	0.60	0.14	1.07	0.16	35	13	56
**B**	2.49	1.32	0.03	2.38	0.08	74	3	60
**C**	2.42	0.47	0.02	0.72	0.06	37	8	50
**D**	1.93	1.19	0.04	1.77	0.02	76	1	60
**E**	3.17	0.54	0.23	1.18	0.44	31	27	62
**F**	3.15	1.02	0.14	2.23	0.26	48	10	56
**G**	3.07	0.40	0.20	0.76	0.47	20	38	53
**H**	2.88	0.75	0.10	1.45	0.17	46	10	49

**Table 6:** Comparison between real and approximated fused area above the reference plane in the cross-section.

Condition	Actual *A_h_* (mm^2^)	Parabolic Approx. (mm^2^)	Mismatch (%)	Circular Approx. (mm^2^)	Mismatch (%)
A	1.07	1.09	1.9	1.13	5.6
B	2.38	2.19	−8.0	2.62	10.1
C	0.72	0.76	5.6	0.78	8.3
D	1.77	1.53	−13.6	1.92	8.5
E	1.18	1.14	−3.4	1.17	−0.8
F	2.23	2.14	−4.0	2.31	3.6
G	0.76	0.82	7.9	0.83	9.2
H	1.45	1.44	−0.7	1.52	4.8

**Table 7:** Average Vickers micro-hardness as a function of the processing conditions; flat deposition.

Condition	Reported Metal		HAZ	
	*HV*_0.2_	Std. dev.	*HV*_0.2_	Std. dev.
A	297	24.2	169	50.3
B	301	19.5	162	36.3
C	311	7.7	175	51.9
D	272	23.9	163	26.1
E	396	24.6	173	51.7
F	410	25.6	164	44.2
G	421	3.5	181	65.2
H	442	4.2	190	69.8

**Table 8:** Average extent of the HAZ as a function of the processing conditions; flat deposition.

Condition	A	B	C	D	E	F	G	H
**HAZ extent (mm)**	2.20	2.05	2.03	2.02	2.54	2.40	2.50	1.90

**Table 9:** Trace surface aspect and corresponding cross-section for deposition over edges.

Condition	Trace Aspect	Cross-Section
**A**	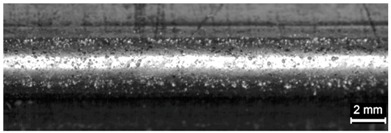	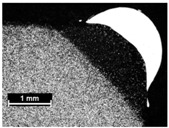
**B**	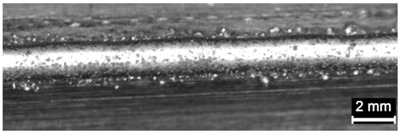	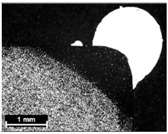
**C**	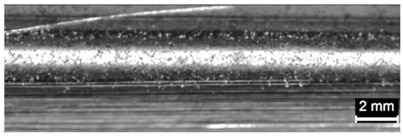	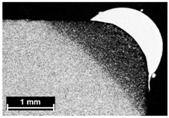
**D**	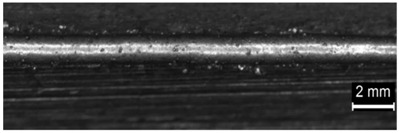	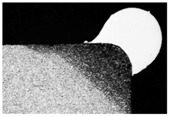
**E**	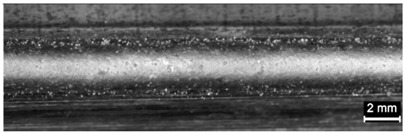	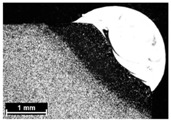
**F**	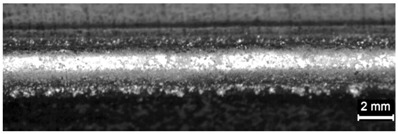	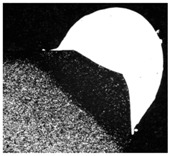
**G**	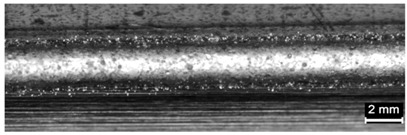	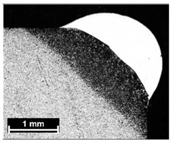
**H**	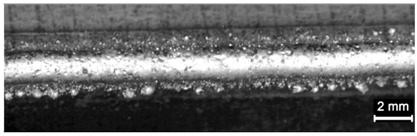	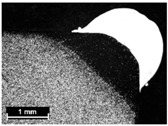

**Table 10:** Average geometrical responses in the cross section for deposition over edges.

Condition	*h*_1_ (mm)	*h*_2_ (mm)	*A_h_* (mm^2^)	*A_d_* (mm^2^)	Dilution (%)	*η* (%)
**A**	0.35	0.39	0.84	0.41	33	44
**B**	0.94	0.71	1.74	0.15	8	44
**C**	0.27	0.32	0.55	0.33	38	38
**D**	0.67	0.57	0.90	0.06	6	30
**E**	0.41	0.34	0.98	1.35	58	51
**F**	0.87	0.62	2.18	0.38	15	55
**G**	0.30	0.38	0.69	0.71	51	48
**H**	0.57	0.58	1.39	0.27	16	47

**Table 11:** Average Vickers micro-hardness as a function of the processing conditions; edge deposition.

Condition	Reported Metal		HAZ	
	*HV*_0.2_	Std. dev.	*HV*_0.2_	Std. dev.
A	527	10.9	177	42.2
B	597	27.5	182	56.2
C	518	5.2	179	51.4
D	589	19.0	194	77.5
E	435	12.4	172	43.8
F	576	11.0	167	37.3
G	438	6.2	187	56.8
H	440	65.7	186	44.7
